# Design, synthesis, in silico studies and biological evaluation of 5-((*E*)-4-((*E*)-(substituted aryl/alkyl)methyl)benzylidene)thiazolidine-2,4-dione derivatives

**DOI:** 10.1186/s13065-020-00678-2

**Published:** 2020-03-31

**Authors:** Harsh Kumar, Aakash Deep, Rakesh Kumar Marwaha

**Affiliations:** 1 Department of Pharmaceutical Sciences, Maharshi Dayanand University, Rohtak, Haryana 124001 India; 2 Department of Pharmaceutical Sciences, Chaudhary Bansi Lal University, Bhiwani, Haryana 127021 India

**Keywords:** Thiazolidin-2,4-dione, Synthesis, Antimicrobial, Antioxidant, Characterization

## Abstract

**Background:**

Looking at the extensive biological potential of thiazolidine-2,4-dione (TZD) moiety, a new series of thiazolidine-2,4-dione analogues was synthesized. Different spectral techniques (^1^H-NMR, IR, MS etc.) were used to confirm the chemical structures of the synthesized analogues. These synthesized compounds were screened for their antioxidant and antimicrobial potential.

**Results and discussion:**

The antimicrobial screening was carried out against selected strains of fungi and bacteria using serial tube dilution method. The antioxidant potential was assessed using stable 2,2-diphenyl-1-picrylhydrazyl (DPPH) free radical scavenging method. Further, the interaction between synthesized thiazolidine-2,4-dione compounds and DNA gyrase was explored using molecular docking studies. Various ADME parameters were also studied to evaluate the drug likeness of the synthesized compounds.

**Conclusion:**

In antimicrobial evaluation, the compounds **4**, **9**, **11**, **12, 13**, **15** and **16** displayed promising activity against selected strains of microbes. Antioxidant evaluation found compound **6** having IC_50_ = 9.18 μg/mL to be the most potent compound in the series. The molecular docking study revealed compounds **4 (**dock score = − 4.73) and **7** (dock score = − 4.61) with decent docking score, possess good interaction inside the ATP binding pocket of DNA gyrase and therefore can be used as lead structure for further optimizing into potent antimicrobial molecule.
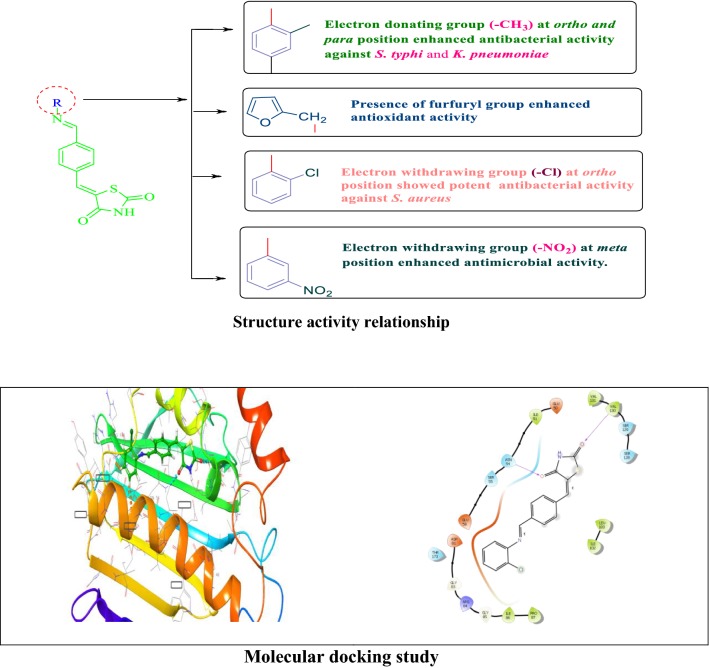

## Introduction

**Fig. 1:**
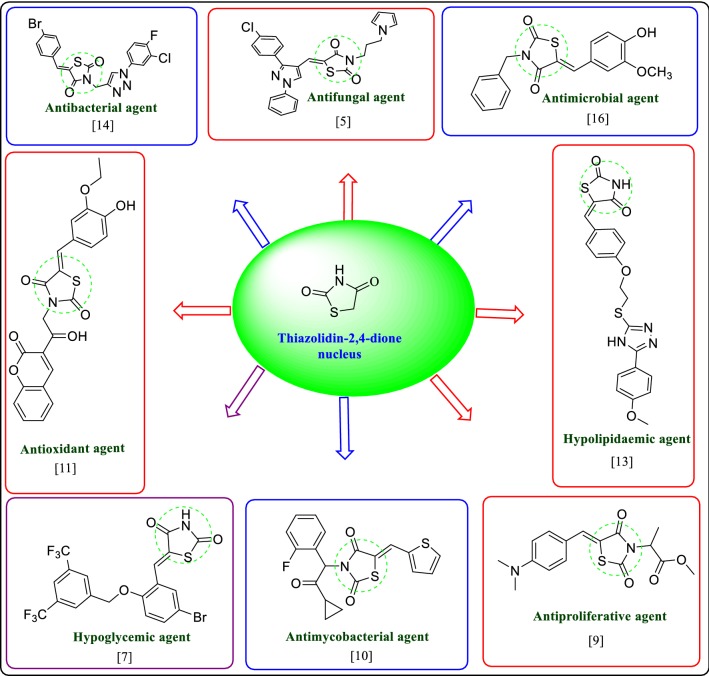
Biological potential of thiazolidin-2,4-dione moiety

The increasing rate of microbial infection and development of drug resistance amongst different microbial strains are the major cause of worry for human life worldwide [1]. Some of these resistant strains, such as multidrug resistant *Staphylococcus aureus* (MRSA) and vancomycin-resistant *enterococci* (VRE) are proficient of surviving the effects of most, if not all, antibiotics currently in use [2]. Emergence of new infectious ailments and development of multidrug resistance are amongst the biggest hurdles in the treatment of microbial infections and therefore imposes the finding of newer antimicrobial compounds [3]. Small heterocyclic rings having sulfur and nitrogen atoms like thiazolidine-2,4-dione (TZD) have been under study for a long time due to their synthetic variety and therapeutic relevance [4]. The TZD moiety is reported to possess extensive biological potential such as antifungal [5], analgesic, anti-inflammatory [6], hypoglycemic [7], antimalarial [8], antiproliferative [9], antitubercular [10], antioxidant [11], antiviral [12], hypolipidemic [13] and antibacterial [14–16] etc. The biological potential of TZD moiety is displayed in Fig. 1.

Expressive view of possible drug-receptor interaction can be a new rational method for drug design which can be explored using molecular docking studies. DNA (Deoxyribonucleic acid) gyrase is a vital enzyme of topoisomerases class that are involved in the regulation of topological transitions of DNA by the formation of negative supercoils. It is also involved in replication and transcription processes. Its inhibition causes DNA disruption which ultimately leads to cell death [17].

Poor pharmacokinetic properties of the drug molecules like absorption, distribution, metabolism and excretion (ADME) are amongst the major causes of failure during drug development process [18]. ADME (absorption, distribution, metabolism and excretion) properties are the critical determinants for the clinical success of the drug molecule which otherwise can be withdrawn from the market due to unexpected toxicity leading huge financial loss [19]. These studies can also help in optimizing a chemical compound with a certain pharmacological or biological activity to be orally active drug in humans [20].

Based on the data attained from literature survey, in the present study we hereby account the synthesis, antioxidant and antimicrobial potentials, molecular docking studies and ADME properties of thiazolidine-2,4-dione derivatives.

## Results and discussion

### Chemistry

**Scheme 1:**
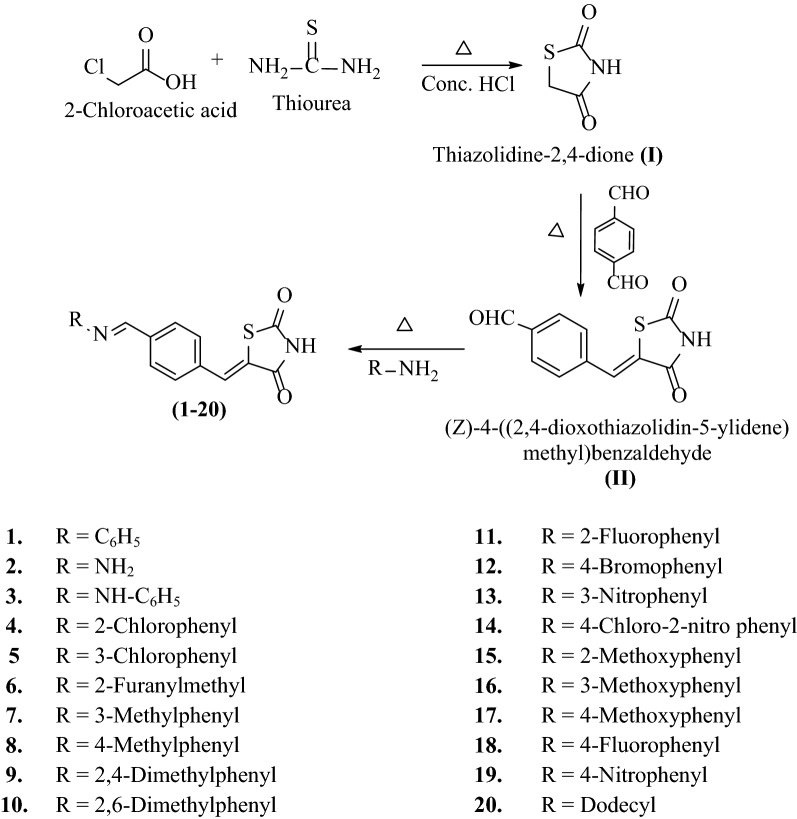
Synthesis of 5-((*E*)-4-((*E*)-(substituted aryl/alkyl)methyl)benzylidene)thiazolidine-2,4-dione derivatives (**1–20**)

The synthesis of TZD derivatives (**1–20**) were accomplished using the synthetic route depicted in Scheme 1. At first, 2-chloroacetic acid was treated with thiourea in conc. HCl to obtain TZD (**I**). Further, the reaction of (**I**) with terephthalaldehyde yielded 4-((2,4-dioxothiazolidin-5-ylidene) methyl) benzaldehyde (**II**). Intermediate-**II** on further treatment with substituted anilines/amines yielded final 5-((*E*)-4-((*E*)-(substituted aryl/alkyl)methyl)benzylidene)thiazolidine-2,4-dione derivatives (**1–20**). The physicochemical characterization and spectral analysis of the synthesized derivatives are given in Table 1. The molecular structures of the synthesized derivatives (**1–20**) were established using elemental analysis and spectral studies [FT-IR (KBr, cm^−1^), ^1^H-NMR (DMSO-*d*_6_, 400 MHz, *δ* ppm) and Mass spectra]. The ^1^H-NMR spectra designated that the presence of multiplet signals between 6.52 and 8.28 *δ* ppm reflected the presence of aromatic protons in synthesized molecules. The presence of singlet(s) between 7.62 and 7.84 *δ* ppm, 7.87–8.80 *δ* ppm and 12.12–12.70 *δ* ppm indicated the presence of –CH=, –CH=N and –NH groups, respectively. The compound **2** exhibited singlet (s) at 1.92 *δ* ppm due to the existence of H of –NH_2_ group. The appearance of singlet (s) at 2.08–2.33 *δ* ppm in compounds **7**, **8**, **9** and **10** revealed the existence of CH_3_ of Ar–CH_3_. The existence of OCH_3_ of Ar–OCH_3_ in the compounds, **15**, **16** and **17** was confirmed by presence of singlet at 3.77–3.85 *δ* ppm. In compound **3** NH of Ar–NH existence was confirmed by appearance of singlet at 10.61 *δ* ppm. The compound **20** displayed multiplet at 1.23**–**1.69 *δ* ppm of CH_2_, triplet at 0.84 *δ* ppm of CH_3_ and multiplet at 3.66 *δ* ppm of CH_2_ adjacent to CH=N due to the existence of dodecyl group. The compound **6** showed doublet signal at 4.77 *δ* ppm of –CH_2_ adjacent to furan ring, doublet signal at 6.30 *δ* ppm of –CH of furan ring at 3rd position, triplet signal at 6.42 *δ* ppm of –CH of furan ring at 4th position and doublet signal at 7.47 *δ* ppm of –CH of furan ring adjacent to O (oxygen) due to the existence of furfuryl group. In case of IR spectrum, the presence of bands at 3437–3286 cm^−1^, 3048–2919 cm^−1^, 3197–3012 cm^−1^, 1615–1548 cm^−1^, 1522–1412 cm^−1^, 1702–1610 cm^−1^, 1747–1614 cm^−1^, and 618–594 cm^−1^ displayed the presence of N–H, C–H (aliphatic), C–H (aromatic), C=C (methylene), C=C (aromatic), C=N, C=O and C–S groups respectively in the synthesized analogues. The absorption bands around 1338–1224 cm^−1^ and 1165–1152 cm^−1^ corresponded to C–N and C–C stretching of compounds, respectively. Compounds **4**, **5** and **14** displayed absorption bands of C–Cl around 774–750 cm^−1^. Mass of the synthesized compounds exhibited M^+^ + 1, M^+^ and M^+^ − 1 peaks.

**Table 1:** The physicochemical, elemental and spectral characteristics of synthesized thiazolidine-2,4-dione derivatives

Compound	Physicochemical and spectral characteristics
	*5*-*((E)*-*4*-*((E)*-*(phenylimino)methyl)benzylidene)thiazolidine*-*2,4*-*dione*: m.p. °C: 260-262; R*f* value: 0.60^b^; % yield: 80; IR (KBr pellets) cm^−1^: 3375.41 (N–H str., thiazolidine ring), 1742.41 (C=O str., thiazolidin-2,4-dione ring), 1696.11 (C=N str., imine group), 1414.1 (C=C str., aromatic ring), 1602.13 (C=C str., methylene group), 3044.78 (C–H str., aromatic ring), 2919.12 (C–H str., aliphatic), 1317.75 (C–N str., thiazolidine ring), 614.77 (C–S bend., thiazolidine ring); ^1^H-NMR (*δ*, DMSO): 6.52–7.75 (m, 9H, Ar–H), 7.77 (s, 1H, –CH=), 8.71(s, 1H, CH=N), 12.49 (s, 1H, NH); M. Formula: C_17_H_12_N_2_O_2_S; MS: *m/z* 308 (M^+^); Elemental analysis (CHN) Theoretical calc: C, 66.22; H, 3.92; N, 9.08 Found: C, 66.25; H, 3.90; N, 9.09
	*5*-*((E)*-*4*-*((E)*-*hydrazonomethyl)benzylidene)thiazolidine*-*2,4*-*dione:* m.p. °C: 220–222; R*f* value: 0.74^c^; % yield: 60; IR (KBr pellets) cm^−1^: 1740.11 (C=O str., thiazolidin-2,4-dione ring), 1682.74 (C=N str., imine group), 1412.68 (C=C str., aromatic ring), 1615.22 (C=C str., methylene group), 3197.24 (C–H str., aromatic ring), 2922.17 (C–H str., aliphatic), 1224.86 (C–N str., thiazolidine ring), 618.11 (C–S bend., thiazolidine ring); ^1^H-NMR (*δ*, DMSO): 6.58–7.79 (m, 4H, Ar–H), 7.81 (s, 1H, –CH=), 8.68(s, 1H, CH=N), 1.92 (s, 2H, NH_2_), 12.49 (s, 1H, NH); M. Formula: C_11_H_9_N_3_O_2_S; Elemental analysis (CHN) Theoretical calc: C, 53.43; H, 3.67; N, 16.99 Found: C, 53.45; H, 3.68; N, 16.95
	*5*-*((E)*-*4*-*((E)*-*(2*-*phenylhydrazono)methyl)benzylidene)thiazolidine*-*2,4*-*dione*: m.p. °C: 308–310; R*f* value: 0.8^d^; % yield: 78; IR (KBr pellets) cm^−1^: 3441.91 (N–H str., phenyl hydrazine group), 3286.35 (N–H str., thiazolidine ring), 1726.16 (C=O str., thiazolidin-2,4-dione ring), 1677.76 (C=N str., imine group), 1413.66 (C=C str., aromatic ring), 1584.3 (C=C str., methylene group), 3043.30 (C–H str., aromatic ring), 1328.68 (C–N str., thiazolidine ring), 613.72 (C–S bend., thiazolidine ring); ^1^H-NMR (*δ*, DMSO): 6.77–7.79 (m, 4H, Ar–H), 7.80 (s, 1H, –CH=), 7.88(s, 1H, CH=N), 10.61 (s, 1H, NH adjacent to phenyl ring), 12.49 (s, 1H, NH of thiazolidine ring); M. Formula: C_17_H_13_N_3_O_2_S; MS: *m/z* 323.9 (M^+^ + 1), 321.9 (M^+^ − 1); Elemental analysis (CHN) Theoretical calc: C, 63.14; H, 4.05; N, 12.99 Found: C, 63.16; H, 4.05; N, 12.98
	*5*-*((E)*-*4*-*((E)*-*((2*-*chlorophenyl)imino)methyl)benzylidene)thiazolidine* -*2,4*-*dione*: m.p. °C: 292–294; R*f* value: 0.71^d^; % yield: 70; IR (KBr pellets) cm^−1^: 3381.37 (N–H str., thiazolidine ring), 1741.97 (C=O str., thiazolidin-2,4-dione ring), 1694.84 (C=N str., imine group), 1505.9 (C=C str., aromatic ring), 1606.57 (C=C str., methylene group), 3044.79 (C–H str., aromatic ring), 2978.2 (C–H str., aliphatic), 1305.25 (C–N str., thiazolidine ring), 606.87 (C–S bend., thiazolidine ring), 750.70 (C–Cl bend., o-substitution on phenyl ring); ^1^H NMR (*δ*, DMSO): 6.77–7.79 (m, 4H, Ar–H), 7.80 (s, 1H, –CH=), 7.88(s, 1H, CH=N), 10.61 (s, 1H, NH adjacent to phenyl ring), 12.49 (s, 1H, NH of thiazolidine ring); M. Formula: C_17_H_11_ClN_2_O_2_S; Elemental analysis (CHN) Theoretical calc: C, 59.56; H, 3.23; N, 8.17 Found: C, 59.60; H, 3.25; N, 8.12
	*5*-*((E)*-*4*-*((E)*-*((3*-*chlorophenyl)imino)methyl)benzylidene) thiazolidine*-*2,4*-*dione*: m.p. °C: 280–282; R*f* value: 0.73^d^; % yield: 75; IR (KBr pellets) cm^−1^: 3381.59 (N–H str., thiazolidine ring), 1741.81 (C=O str., thiazolidin-2,4-dione ring), 1695.1 (C=N str., imine group), 1479.44 (C=C str., aromatic ring), 1596.41 (C=C str., methylene group), 3050.78 (C–H str., aromatic ring), 2978.82 (C–H str., aliphatic), 1308.79 (C–N str., thiazolidine ring), 594.24 (C–S bend., thiazolidine ring), 777.34 (C–Cl bend., m-substitution on phenyl ring); ^1^H NMR (*δ*, DMSO): 7.23–8.07 (m, 8H, Ar–H), 7.81 (s, 1H, –CH=), 8.68 (s, 1H, CH=N), 12.58 (s, 1H, NH); M. Formula: C_17_H_11_ClN_2_O_2_S; MS: *m/z* 341.43 (M^+^ − 1); Elemental analysis (CHN) Theoretical calc: C, 59.56; H, 3.23; N, 8.17 Found: C, 59.62; H, 3.23; N, 8.18
	*5*-*((E)*-*4*-*((E)*-*((furan*-*2*-*ylmethyl)imino)methyl)benzylidene) thiazolidine*-*2,4*-*dione*: m.p. °C: 92–94; R*f* value: 0.71^a^; % yield: 70; IR IR (KBr pellets) cm^−1^: 3328.11 (N–H str., thiazolidine ring), 1694.77 (C=O str., thiazolidin-2,4-dione ring), 1610.48 (C=N str., imine group), 1423.16 (C=C str., aromatic ring), 1548.31 (C=C str., methylene group), 3045.54 (C–H str., aromatic ring), 2975.08 (C–H str., aliphatic), 1306.66 (C–N str., thiazolidine ring), 603.55 (C–S bend., thiazolidine ring), 1159.90 (C–C str.), 1012.66 (C–O–C str., furan ring); ^1^H NMR (*δ*, DMSO): 7.18–7.88 (m, 4H, Ar–H), 7.78 (s, 1H, –CH=), 8.76 (s, 1H, CH=N), 12.48 (s, 1H, NH), 4.77 (d, 2H, –CH_2_ adjacent to furan ring), 6.30 (d, 1H, CH of furan ring at 3^rd^ position), 6.42 (t, 1H, CH of furan ring at 4th position), 7.47 (d, 1H, CH of furan ring adjacent to O); M. Formula: C_16_H_12_N_2_O_3_S; MS: *m/z* 313.16 (M^+^ + 1); Elemental analysis (CHN) Theoretical calc: C, 61.53; H, 3.87; N, 8.97 Found: C, 61.55; H, 3.90; N, 8.95
	*5*-*((E)*-*4*-*((E)*-*(m*-*tolylimino)methyl)benzylidene)thiazolidine*-*2,4*-*dione*: m.p. °C: 90–92; R*f* value: 0.81^a^; % yield: 65; IR (KBr pellets) cm^−1^: 3378.54 (N–H str., thiazolidine ring), 1743.48 (C=O str., thiazolidin-2,4-dione ring), 1691.85 (C=N str., imine group), 1487.15 (C=C str., aromatic ring), 1602.26 (C=C str., methylene group), 3036.12 (C–H str., aromatic ring), 2967.46 (C–H str., aliphatic), 1321.95 (C–N str., thiazolidine ring), 597.53 (C–S bend., thiazolidine ring), 1154.43 (C–C str.); ^1^H NMR (*δ*, DMSO): 7.08–8.06 (m, 8H, Ar–H), 7.62 (s, 1H, –CH=), 8.43 (s, 1H, CH=N), 12.34 (s, 1H, NH), 2.14(s, 3H, CH_3_); M. Formula: C_18_H_14_N_2_O_2_S; MS: *m/z* 320.96 (M^+^ − 1); Elemental analysis (CHN) Theoretical calc: C, 67.06; H, 4.38; N, 8.69 Found: C, 67.09; H, 4.39; N, 8.71
	*5*-*((E)*-*4*-*((E)*-*(p*-*tolylimino)methyl)benzylidene)thiazolidine*-*2,4*-*dione*: m.p. °C: 160–162; R*f* value: 0.84^a^; % yield: 75; IR (KBr pellets) cm^−1^: 3375.37 (N–H str., thiazolidine ring), 1744.26 (C=O str., thiazolidin-2,4-dione ring), 1695.41 (C=N str., imine group), 1507.28 (C=C str., aromatic ring), 1604.31 (C=C str., methylene group), 3162.34 (C–H str., aromatic ring), 3048.39 (C–H str., aliphatic), 1330.13 (C–N str., thiazolidine ring), 607.48 (C–S bend., thiazolidine ring), 1155.49 (C–C str.); ^1^H NMR (*δ*, DMSO): 6.80–8.06 (m, 8H, Ar–H), 7.72 (s, 1H, –CH=), 8.71 (s, 1H, CH=N), 12.32 (s, 1H, NH), 2.33(s, 3H, CH_3_); M. Formula: C_18_H_14_N_2_O_2_S; MS: *m/z* 322.01 (M^+^); Elemental analysis (CHN) Theoretical calc: C, 67.06; H, 4.38; N, 8.69 Found: C, 67.11; H, 4.39; N, 8.65
	*5*-*((E)*-*4*-*((E)*-*((2,4*-*dimethylphenyl)imino)methyl)benzylidene) thiazolidine*-*2,4*-*dione*: m.p. °C: 134–136; R*f* value: 0.81^a^; % yield: 65; IR (KBr cm^−1^): 3398.47 (N–H str., thiazolidine ring), 1744.74 (C=O str., thiazolidin-2,4-dione ring), 1699.13 (C=N str., imine group), 1498.40 (C=C str., aromatic ring), 1601.47 (C=C str., methylene group), 3012.68 (C–H str., aromatic ring), 2973.04 (C–H str., aliphatic), 1291.9 (C–N str., thiazolidine ring), 605.41 (C–S bend., thiazolidine ring), 1152.01 (C–C str.); ^1^H NMR (*δ*, DMSO): 7.02–8.06 (m, 7H, Ar–H), 7.82 (s, 1H, –CH=), 8.58 (s, 1H, CH=N), 12.12 (s, 1H, NH), 2.28(s, 3H, CH_3_ o-position), 2.29(s, 3H, CH_3_ p-position); M. Formula: C_19_H_16_N_2_O_2_S; MS: *m/z* 334.99 (M^+^ − 1); Elemental analysis (CHN) Theoretical calc: C, 67.84; H, 4.79; N, 8.33 Found: C, 67.87; H, 4.79; N, 8.36
	*5*-*((E)*-*4*-*((E)*-*((2,6*-*dimethylphenyl)imino)methyl)benzylidene) thiazolidine*-*2,4*-*dione*: m.p. °C: 100–102; R*f* value: 0.73^a^; % yield: 60; IR (KBr cm^−1^): 3394.22 (N–H str., thiazolidine ring), 1744.97 (C=O str., thiazolidin-2,4-dione ring), 1702.53 (C=N str., imine group), 1470.88 (C=C str., aromatic ring), 1602.92 (C=C str., methylene group), 3032.72 (C–H str., aromatic ring), 2971.09 (C–H str., aliphatic), 1294.35 (C–N str., thiazolidine ring), 606.56 (C–S bend., thiazolidine ring), 1159.9 (C–C str.); ^1^H NMR (*δ*, DMSO): 6.77–8.10 (m, 7H, Ar–H), 7.84 (s, 1H, –CH=), 8.42 (s, 1H, CH=N), 12.32 (s, 1H, NH), 2.08(s, 6H, CH_3 o_-position); M. Formula: C_19_H_16_N_2_O_2_S; MS: *m/z* 334.98 (M^+^ − 1), 337 (M^+^ + 1); Elemental analysis (CHN) Theoretical calc: C, 67.84; H, 4.79; N, 8.33 Found: C, 67.85; H, 4.79; N, 8.36
	*5*-*((E)*-*4*-*((E)*-*((2*-*fluorophenyl)imino)methyl)benzylidene) thiazolidine*-*2,4*-*dione*: m.p. °C: 108–110; R*f* value: 0.69^a^; % yield: 75; IR (KBr cm^−1^): 3429.95 (N–H str., thiazolidine ring), 1745.15 (C=O str., thiazolidin-2,4-dione ring), 1695.31 (C=N str., imine group), 1492.16 (C=C str., aromatic ring), 1604.74 (C=C str., methylene group), 3051.62 (C–H str., aromatic ring), 2979.31 (C–H str., aliphatic), 1323.63 (C–N str., thiazolidine ring), 606.47 (C–S bend., thiazolidine ring), 1158.78 (C–C str.), 1009.17 (C-F bend., o-substitution on phenyl ring); ^1^H NMR (*δ*, DMSO): 7.26–7.76 (m, 8H, Ar–H), 7.83 (s, 1H, –CH=), 8.68 (s, 1H, CH=N), 12.51 (s, 1H, NH); M. Formula: C_17_H_11_N_2_O_2_SF; MS: *m/z* 327.05 (M^+^ + 1); Elemental analysis (CHN) Theoretical calc: C, 62.57; H, 3.40; N, 8.58 Found: C, 62.60; H, 3.41; N, 8.60
	*5*-*((E)*-*4*-*((E)*-*((3*-*bromophenyl)imino)methyl)benzylidene) thiazolidine*-*2,4*-*dione*: m.p. °C: 98–100; R*f* value: 0.82^a^; % yield: 80; IR (KBr cm^−1^): 3437.95 (N–H str., thiazolidine ring), 1744.75 (C=O str., thiazolidin-2,4-dione ring), 1698.27 (C=N str., imine group), 1484.71 (C=C str., aromatic ring), 1606.87 (C=C str., methylene group), 3052.46 (C–H str., aromatic ring), 2976.98 (C–H str., aliphatic), 1328.18 (C–N str., thiazolidine ring), 606.38 (C–S bend., thiazolidine ring), 1161.85 (C–C str.), 637.54 (C–Br bend., p-substitution on phenyl ring); ^1^H NMR (*δ*, DMSO): 7.03–8.12 (m, 8H, Ar–H), 7.78 (s, 1H, –CH=), 8.72 (s, 1H, CH=N), 12.64 (s, 1H, NH); M. Formula: C_17_H_11_N_2_O_2_SBr; Elemental analysis (CHN) Theoretical calc: C, 52.73; H, 2.86; N, 7.23 Found: C, 52.75; H, 2.87; N, 7.23
	*5*-*((E)*-*4*-*((E)*-*((3*-*nitrophenyl)imino)methyl)benzylidene)thiazolidine*-*2,4*-*dione*: m.p. °C: 128–130; R*f* value: 0.79^a^; % yield: 80; IR (KBr cm^−1^): 3379.48 (N–H str., thiazolidine ring), 1747.82 (C=O str., thiazolidin-2,4-dione ring), 1700.55 (C=N str., imine group), 1522.68 (C=C str., aromatic ring), 1600.01 (C=C str., methylene group), 3034.11 (C–H str., aromatic ring), 2928.16 (C–H str., aliphatic), 1347.29 (C–N str., thiazolidine ring), 606.91 (C–S bend., thiazolidine ring), 1153.91 (C–C str.), 1208.69 (N–O str., m-substitution on phenyl ring), 1413.00 (N=O str., m-substitution on phenyl ring); ^1^H NMR (*δ*, DMSO): 7.23–8.09 (m, 8H, Ar–H), 7.77 (s, 1H, –CH=), 8.80 (s, 1H, CH=N), 12.67 (s, 1H, NH); M. Formula: C_17_H_11_N_3_O_4_S; MS: *m/z* 352.04 (M^+^ − 1); Elemental analysis (CHN) Theoretical calc: C, 57.79; H, 3.14; N, 11.89 Found: C, 57.82; H, 3.15; N, 11.91
	*5*-*((E)*-*4*-*((E)*-*((4*-*chloro*-*2*-*nitrophenyl)imino)methyl)benzylidene) thiazolidine*-*2,4*-*dione*: m.p. °C: 92–94; R*f* value: 0.81^a^; % yield: 75; IR (KBr cm^−1^): 3356. 19 (N–H str., thiazolidine ring), 1747.59 (C=O str., thiazolidin-2,4-dione ring), 1700.69 (C=N str., imine group), 1502.15 (C=C str., aromatic ring), 1602.45 (C=C str., methylene group), 3030.51 (C–H str., aromatic ring), 2926.60 (C–H str., aliphatic), 1338.08 (C–N str., thiazolidine ring), 607.48 (C–S bend., thiazolidine ring), 1160.38 (C–C str.), 1249.21 (N–O str., o-substitution on phenyl ring), 1456.19 (N=O str., o-substitution on phenyl ring), 764.11 (C–Cl bend., p-substitution on phenyl ring); ^1^H NMR (*δ*, DMSO): 7.03–8.01 (m, 7H, Ar–H), 7.73 (s, 1H, –CH=), 8.03 (s, 1H, CH=N), 12.70 (s, 1H, NH); M. Formula: C_17_H_10_N_3_O_4_SCl; Elemental analysis (CHN) Theoretical calc: C, 52.65; H, 2.60; N, 10.84 Found: C, 52.67; H, 2.60; N, 10.87
	*5*-*((E)*-*4*-*((E)*-*((2*-*methoxyphenyl)imino)methyl)benzylidene) thiazolidine*-*2,4*-*dione*: m.p. °C: 104–106; R*f* value: 0.81^a^; % yield: 70; IR (KBr cm^−1^): 3396.28 (N–H str., thiazolidine ring), 1744.31 (C=O str., thiazolidin-2,4-dione ring), 1702.51 (C=N str., imine group), 1504.09 (C=C str., aromatic ring), 1602.96 (C=C str., methylene group), 3050.93 (C–H str., aromatic ring), 2930.71 (C–H str., aliphatic), 1293.57 (C–N str., thiazolidine ring), 606.45 (C–S bend., thiazolidine ring), 1161.16 (C–C str.), 1018.82 (O–CH_3_ str., o-substitution on phenyl ring); ^1^H NMR (*δ*, DMSO): 6.75–7.72 (m, 8H, Ar–H), 7.78 (s, 1H, –CH=), 8.58 (s, 1H, CH=N), 12.61 (s, 1H, NH), 3.77 (s, 3H, OCH_3_); M. Formula: C_18_H_14_N_2_O_3_S; MS: *m/z* 339.12 (M^+^ + 1); Elemental analysis (CHN) Theoretical calc: C, 63.89; H, 4.17; N, 8.28 Found: C, 63.91; H, 4.17; N, 8.33
	*5*-*((E)*-*4*-*((E)*-*((3*-*methoxyphenyl)imino)methyl)benzylidene) thiazolidine*-*2,4*-*dione*: m.p. °C: 176–178; R*f* value: 0.82^a^; % yield: 65; IR (KBr cm^−1^): 3380.83 (N–H str., thiazolidine ring), 1742.00 (C=O str., thiazolidin-2,4-dione ring), 1696.51 (C=N str., imine group), 1498.71 (C=C str., aromatic ring), 1601.06 (C=C str., methylene group), 2926.44 (C–H str., aliphatic), 1321.31 (C–N str., thiazolidine ring), 605.30 (C–S bend., thiazolidine ring), 1155.65 (C–C str.), 1029.32 (O–CH_3_ str., m-substitution on phenyl ring); ^1^H NMR (*δ*, DMSO): 6.58–8.05 (m, 8H, Ar–H), 7.73 (s, 1H, –CH=), 8.68 (s, 1H, CH=N), 12.32 (s, 1H, NH), 3.85(s, 3H, OCH_3_); M. Formula: C_18_H_14_N_2_O_3_S; MS: *m/z* 338.8 (M^+^ + 1); Elemental analysis (CHN) Theoretical calc: C, 63.89; H, 4.17; N, 8.28 Found: C, 63.92; H, 4.17; N, 8.31
	*5*-*((E)*-*4*-*((E)*-*((4*-*methoxyphenyl)imino)methyl)benzylidene) thiazolidine*-*2,4*-*dione*: m.p. °C: 130–132; R*f* value: 0.77^a^; % yield: 75; IR (KBr cm^−1^): 3444.26 (N–H str., thiazolidine ring), 1743.88 (C=O str., thiazolidin-2,4-dione ring), 1702.79 (C=N str., imine group), 1505.14 (C=C str., aromatic ring), 1614.33 (C=C str., methylene group), 3065.92 (C–H str., aromatic ring), 2926.15 (C–H str., aliphatic), 1291.79 (C–N str., thiazolidine ring), 606.22 (C–S bend., thiazolidine ring), 1155.22 (C–C str.), 1024.26 (O–CH_3_ str., o-substitution on phenyl ring); ^1^H NMR (*δ*, DMSO): 6.98–8.04 (m, 8H, Ar–H), 7.78 (s, 1H, –CH=), 8.71 (s, 1H, CH=N), 12.42 (s, 1H, NH), 3.78(s, 3H, OCH_3_); M. Formula: C_18_H_14_N_2_O_3_S; MS: *m/z* 338.98 (M^+^ + 1); Elemental analysis (CHN) Theoretical calc: C, 63.89; H, 4.17; N, 8.28 Found: C, 63.93; H, 4.17; N, 8.30
	*5*-*((E)*-*4*-*((E)*-*((4*-*fluorophenyl)imino)methyl)benzylidene) thiazolidine*-*2,4*-*dione*: m.p. °C: 104–106; R*f* value: 0.83^a^; % yield: 70; IR (KBr cm^−1^): 3431.86 (N–H str., thiazolidine ring), 1743.59 (C=O str., thiazolidin-2,4-dione ring), 1697.01 (C=N str., imine group), 1501.17 (C=C str., aromatic ring), 1612.03 (C=C str., methylene group), 3039.20 (C–H str., aromatic ring), 2925.63 (C–H str., aliphatic), 1292.50 (C–N str., thiazolidine ring), 604.82 (C–S bend., thiazolidine ring), 1152.02 (C–C str.), 1012.73 (C-F str., p-substitution on phenyl ring); ^1^H NMR (*δ*, DMSO): 6.77–7.78 (m, 8H, Ar–H), 7.77 (s, 1H, –CH=), 7.87 (s, 1H, CH=N), 12.60 (s, 1H, NH); M. Formula: C_17_H_11_N_2_O_2_SF; MS: *m/z* 324.85 (M^+^ − 1); Elemental analysis (CHN) Theoretical calc: C, 62.57; H, 3.40; N, 8.58 Found: C, 62.58; H, 3.40; N, 8.59
	*5*-*((E)*-*4*-*((E)*-*((4*-*nitrophenyl)imino)methyl)benzylidene)thiazolidine*-*2,4*-*dione*: m.p. °C: 118–120; R*f* value: 0.83^a^; % yield: 75; IR (KBr cm^−1^): 3369.56 (N–H str., thiazolidine ring), 1743.23 (C=O str., thiazolidin-2,4-dione ring), 1698.03 (C=N str., imine group), 1503.33 (C=C str., aromatic ring), 1601.16 (C=C str., methylene group), 3052.03 (C–H str., aromatic ring), 2925.51 (C–H str., aliphatic), 1310.97 (C–N str., thiazolidine ring), 608.61 (C–S bend., thiazolidine ring), 1163.00 (C–C str.), 1212.91 (N–O str., p-substitution on phenyl ring), 1412.67 (N=O str., p-substitution on phenyl ring); ^1^H NMR (*δ*, DMSO): 6.60–8.28 (m, 8H, Ar–H), 7.78 (s, 1H, –CH=), 8.72 (s, 1H, CH=N), 12.62 (s, 1H, NH); M. Formula: C_17_H_11_N_3_O_4_S; Elemental analysis (CHN) Theoretical calc: C, 57.79; H, 3.14; N, 11.89 Found: C, 57.80; H, 3.14; N, 11.88
	*5*-*((E)*-*4*-*((E)*-*(dodecylimino)methyl)benzylidene)thiazolidine*-*2,4*-*dione*: m.p. °C: 106–108; R*f* value: 0.43^a^; % yield: 60; IR (KBr cm^−1^): 3339.52 (N–H str., thiazolidine ring), 1699.02 (C=O str., thiazolidin-2,4-dione ring), 1608.43 (C=N str., imine group), 1461.90 (C=C str., aromatic ring), 1562.34 (C=C str., methylene group), 3047.23 (C–H str., aromatic ring), 2923.03 (C–H str., aliphatic), 1296.98 (C–N str., thiazolidine ring), 608.00 (C–S bend., thiazolidine ring), 1165.06 (C–C str.); ^1^H NMR (*δ*, DMSO): 7.28–7.98 (m, 4H, Ar–H), 7.78 (s, 1H, –CH=), 8.35 (s, 1H, CH=N), 12.62 (s, 1H, NH), 1.23–1.69 (m, 20H, CH_2_), 0.84 (t, *J* = 9.00 Hz, 3H, CH_3_), 3.66 (m, 2H, CH_2_ adjacent to CH=N); M. Formula: C_23_H_32_N_2_O_2_S; MS: *m/z* 401.12 (M^+^ + 1), 399.09 (M^+^ − 1); Elemental analysis (CHN) Theoretical calc: C, 68.96; H, 8.05; N, 6.99 Found: C, 68.97; H, 8.05; N, 6.98

TLC mobile phase = ^a^ Chloroform:Methanol: 9:1

^b^Chloroform:Toluene:GAA: 1:1:0.1

^c^Ethyl Acetate

^d^Methanol:Toluene:GAA: 1:1:0.1

### Antimicrobial activity

**Fig. 2:**
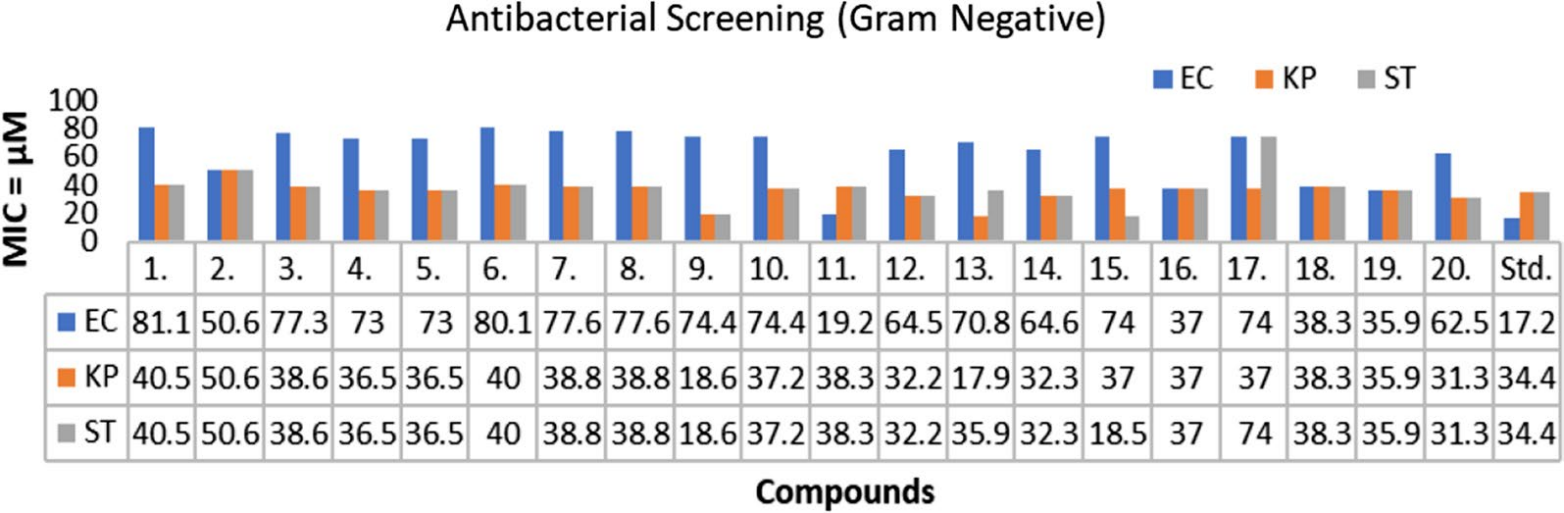
Antibacterial screening results against Gram negative species

**Fig. 3:**
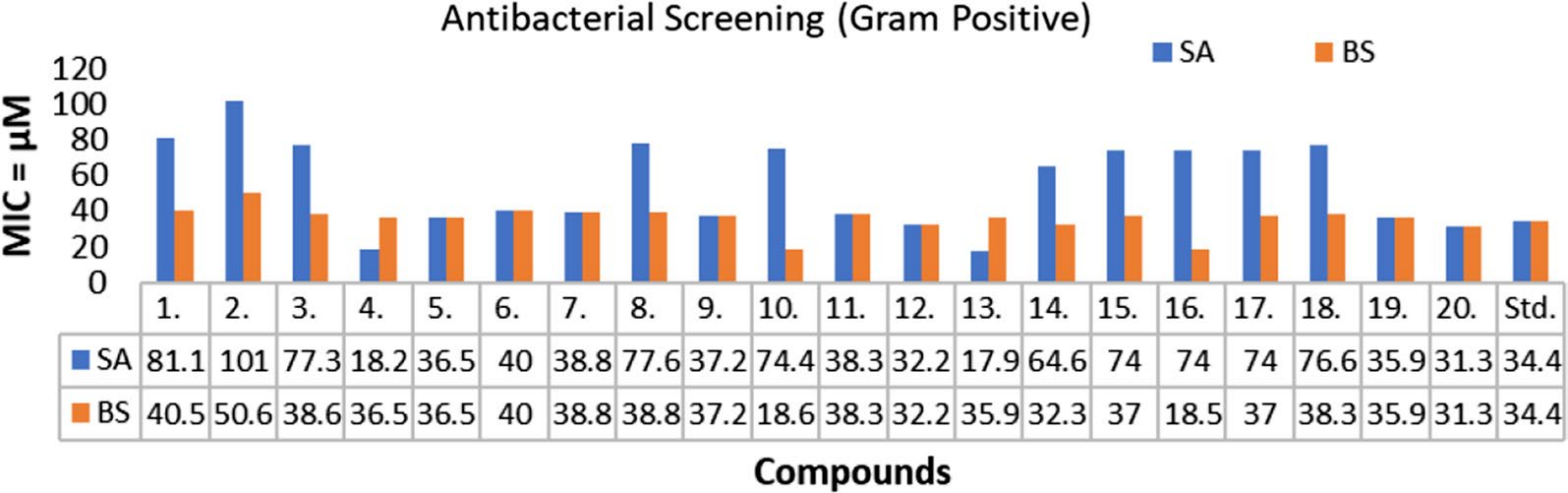
Antibacterial screening results against Gram positive species

**Fig. 4:**
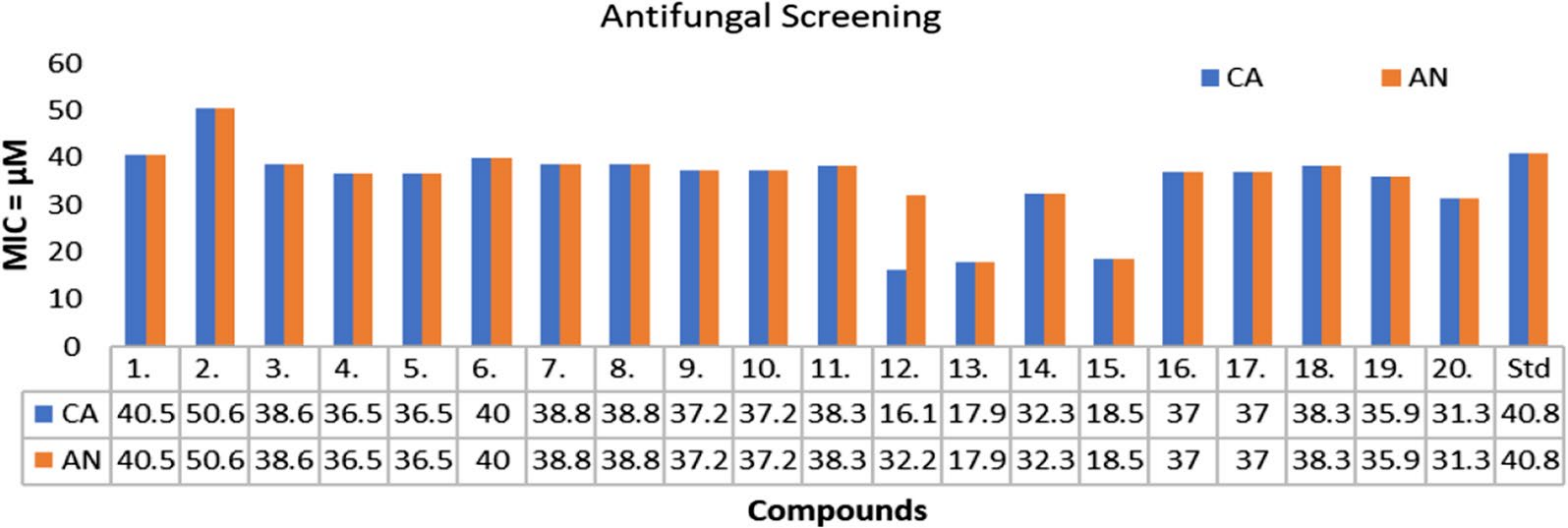
Antifungal screening results against fungal species

The in vitro antimicrobial screening studies of the synthesized TZD derivatives was evaluated by serial tube dilution procedure (Table 2, Figs. 2, 3 and 4). The antibacterial screening outcomes revealed that compounds **13** and **4** were moderately active against *S. aureus* with MIC (Minimum inhibitory concentration) value of 17.9 µM and 18.2 µM, respectively. Further screening revealed that compounds **16** and **10** were moderately active against *B. subtilis* with MIC value of 18.5 µM and 18.6 µM, respectively. Compound **13** (MIC = 17.9 µM) and Compound **9** (MIC = 18.6 µM) were found to be effective against *K. pneumoniae*. Compound **11** (MIC = 19.2 µM) possessed good activity against *E. coli*. Compound **5** (MIC = 18.5 µM) and compound **10** (MIC= 18.6 µM) exhibited promising activity against *S. typhi*. The antifungal screening results revealed that the compounds **13** (MIC = 17.9 µM) and **12** (MIC= 16.1 µM) had good activity against *A. niger* and *C. albicans* respectively. The antibacterial screening results were found to be comparable with the standard drug (cefadroxil), whereas antifungal results of synthesized molecules exhibited superior activity against both the fungal strains, i.e. *A. niger* and *C. albicans* except compound **2** in comparison to the standard drug (fluconazole). So, these synthesized compounds can be taken as lead structures and may further be optimized to yield new antimicrobial agents with better activity.

**Table 2:** In vitro antimicrobial activity of the synthesized compounds

Comp.	Antimicrobial screening (MIC = µM)						
	*SA*	*BS*	*EC*	*KP*	*ST*	*CA*	*AN*
**1**	81.1	40.5	81.1	40.5	40.5	40.5	40.5
**2**	101.2	50.6	50.6	50.6	50.6	50.6	50.6
**3**	77.3	38.6	77.3	38.6	38.6	38.6	38.6
**4**	*18.2*	36.5	73.0	36.5	36.5	36.5	36.5
**5**	36.5	36.5	73.0	36.5	36.5	36.5	36.5
**6**	40.0	40.0	80.1	40.0	40.0	40.0	40.0
**7**	38.8	38.8	77.6	38.8	38.8	38.8	38.8
**8**	77.6	38.8	77.6	38.8	38.8	38.8	38.8
**9**	37.2	37.2	74.4	*18.6*	*18.6*	37.2	37.2
**10**	74.4	*18.6*	74.4	37.2	37.2	37.2	37.2
**11**	38.3	38.3	*19.2*	38.3	38.3	38.3	38.3
**12**	32.2	32.2	64.5	32.2	32.2	*16.1*	32.2
**13**	*17.9*	35.9	70.8	*17.9*	35.9	*17.9*	*17.9*
**14**	64.6	32.3	64.6	32.3	32.3	32.3	32.3
**15**	74.0	37.0	74.0	37.0	*18.5*	18.5	*18.5*
**16**	74.0	*18.5*	37.0	37.0	37.0	37.0	37.0
**17**	74.0	37.0	74.0	37.0	74.0	37.0	37.0
**18**	76.6	38.3	38.3	38.3	38.3	38.3	38.3
**19**	35.9	35.9	35.9	35.9	35.9	35.9	35.9
**20**	31.3	31.3	62.5	31.3	31.3	31.3	31.3
Cefadroxil	34.4	34.4	17.2	34.4	34.4	–	–
Fluconazole	–	–	–	–	–	40.8	40.8

Italic signifies best MIC values against selected microbial strains

SA: *Staphylococcus aureus*, BS: *Bacillus subtilis*, EC: *Escherichia coli*, KP: *Klebsiella pneumoniae*, ST: *Salmonella typhi*; CA: *Candida albicans*, AN: *Aspergillus niger*

### Antioxidant evaluation

**Fig. 5:**
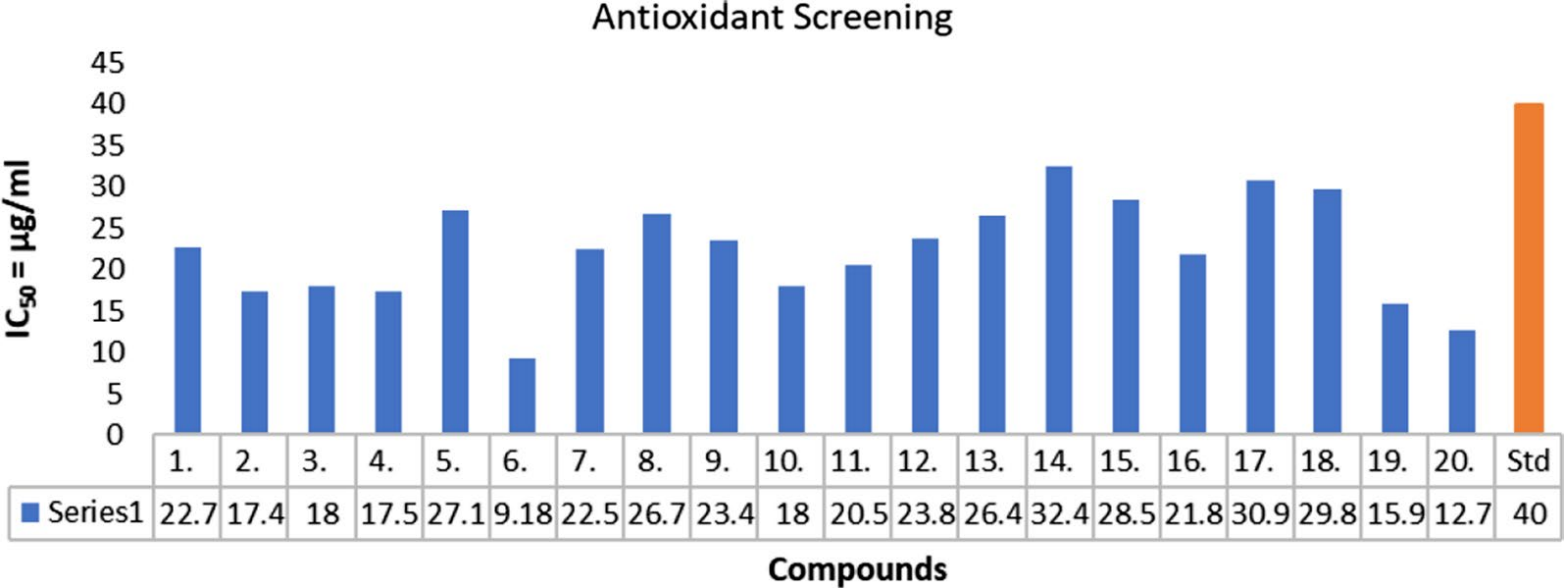
Antioxidant evaluation of synthesized compounds using ascorbic acid as standard

The antioxidant efficacy of the newly synthesized derivatives was assessed by applying DPPH (2,2-diphenyl-1-picrylhydrazyl) radical scavenging method [21] using ascorbic acid as standard drug. 2,2-diphenyl-1-picrylhydrazyl radical is a stable free radical which converts into a stable diamagnetic molecule by accepting an electron or a hydrogen radical. A strong absorption band at 517 nm is observed by methanolic solution of DPPH radical due to presence of an odd electron. DPPH radical reacts with appropriate reducing agent to produce new bond, which leads to change in the color of the solution. As the concentration of antioxidant compound increases in the solution, more electrons are taken up by the DPPH radical from the antioxidant molecules leading to loss in the color intensity of the solution. Such reactivity has been used to test the ability of compounds that can act as free radical scavengers. Reduction of the DPPH radicals has been monitored at 517 nm absorbance spectrophotometrically indicated by decrease in the intensity of color (Purple color) [22]. The IC_50_ value in μg/mL was calculated for all the synthesized compounds. The antioxidant assay revealed all the synthesized compounds to be more potent than the standard drug. Further from the tested antioxidant results, compound 6 (IC_50_ = 9.18 μg/mL) was found to be the most active and showed prominent activity results compared to standard drug. Results are displayed in Table 3 and Fig. 5.

**Table 3:** In vitro antioxidant activity of the synthesized compounds

Comp.	Antioxidant activity (IC_50_ = µg/ml)
1	22.70
**2**	17.37
**3**	18.02
**4**	17.46
**5**	27.11
**6**	*09.18*
**7**	22.45
**8**	26.65
**9**	23.44
**10**	17.96
**11**	20.51
**12**	23.78
**13**	26.44
**14**	32.43
**15**	28.47
**16**	21.81
**17**	30.87
**18**	29.75
**19**	15.93
**20**	12.67
Ascorbic Acid	40

Italic signifies best IC_50_ value (antioxidant activity)

### Molecular docking results

DNA gyrase, a member of topoisomerases type II family has two genes i.e. GyrA (DNA gyrase subunit A) and GyrB (DNA gyrase subunit B), which controls the topological state of DNA in cells [23]. During replication process DNA gyrase is required for maintenance of DNA topology during supercoiling of DNA through coupling of ATP (Adenosine triphosphate) hydrolysis by the GyrB subunit. DNA gyrase enzyme inhibition results in disruption of DNA synthesis in bacterial species leading to bacterial cell death [24].

Molecular docking study was carried out to analyze the binding affinity of the synthesized compounds with ATP binding pocket of DNA gyrase enzyme. The molecular docking study was carried out on GLIDE docking program. All the synthesized compounds were docked in the active site of the *S. aureus* GyrB ATPase domain (PDB: 3U2D) co-crystallized with 08B ligand. Binding affinity of compounds was compared using ATP as docking control. The results were investigated by comparing the docking score obtained from GLIDE (Table 4).

**Table 4:** In silico docking score of the synthesized compounds with 3U2D protein

Comp.	Docking score
1	**− **2.967
**2**	**− **4.07
**3**	**− **2.724
**4**	**− ***4.73*
**5**	**− **4.221
**6**	**− **4.199
**7**	**− ***4.61*
**8**	**− **4.354
**9**	**− **3.184
**10**	**− **2.436
**11**	**− **3.24
**12**	**− **3.859
**13**	**− **3.198
**14**	**− **3.474
**15**	**− **3.663
**16**	**− **3.987
**17**	**− **4.601
**18**	**− **2.818
**19**	**− **4.043
**20**	**− **3.098
Ofloxacin	**− **5.107

Italic signifies best docking score

**Fig. 6:**
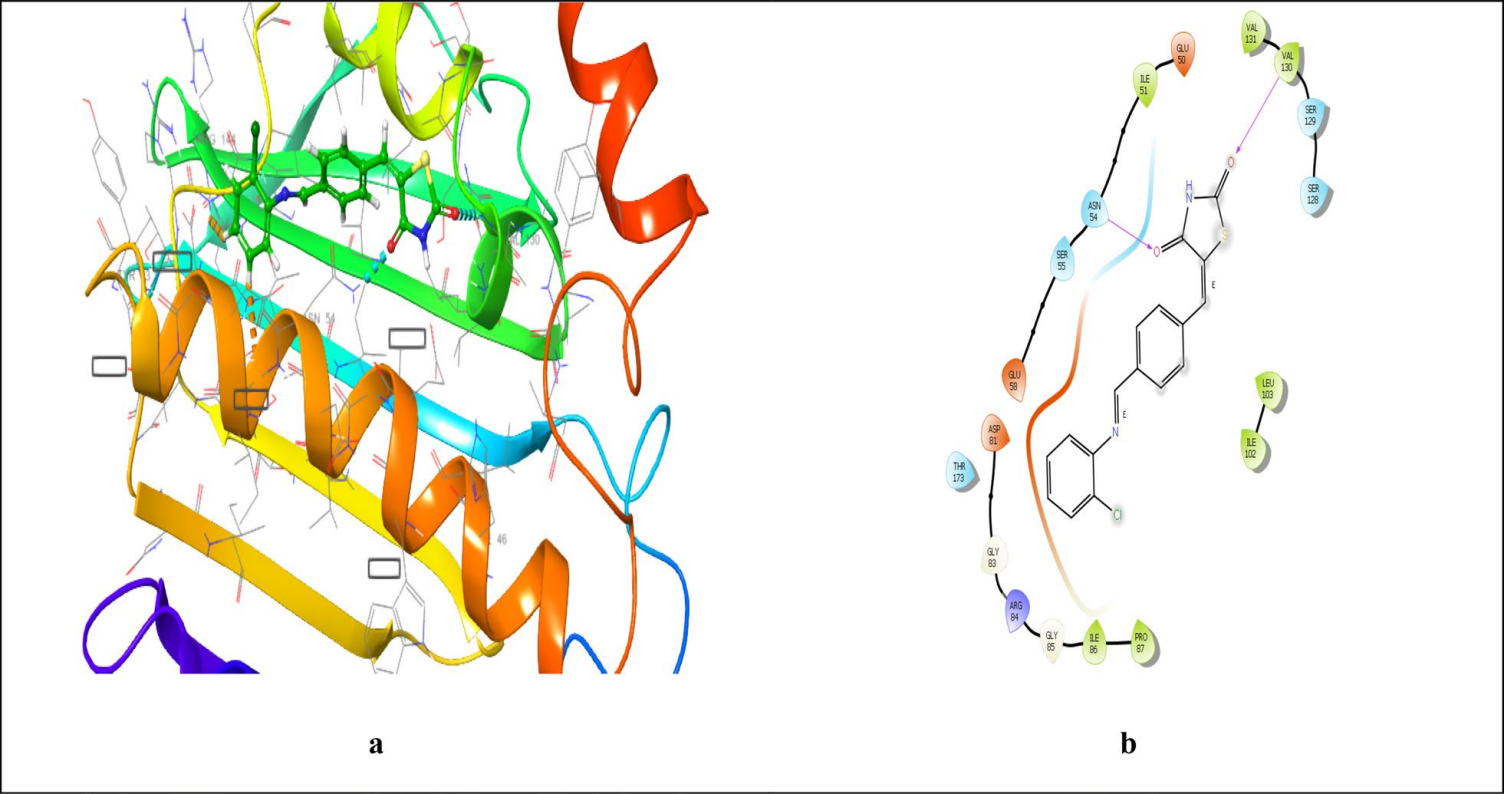
Interaction of compound **4** and 4-bromo-5-methyl-*N*-[1-(3-nitropyridin-2-yl)piperidin-4-yl]-1*H*-pyrrole-2-carboxamide within the active pocket of *S. aureus* GyrB ATPase domain protein and interacting amino acid in 3D (**a**) and 2D (**b**) view

**Fig. 7:**
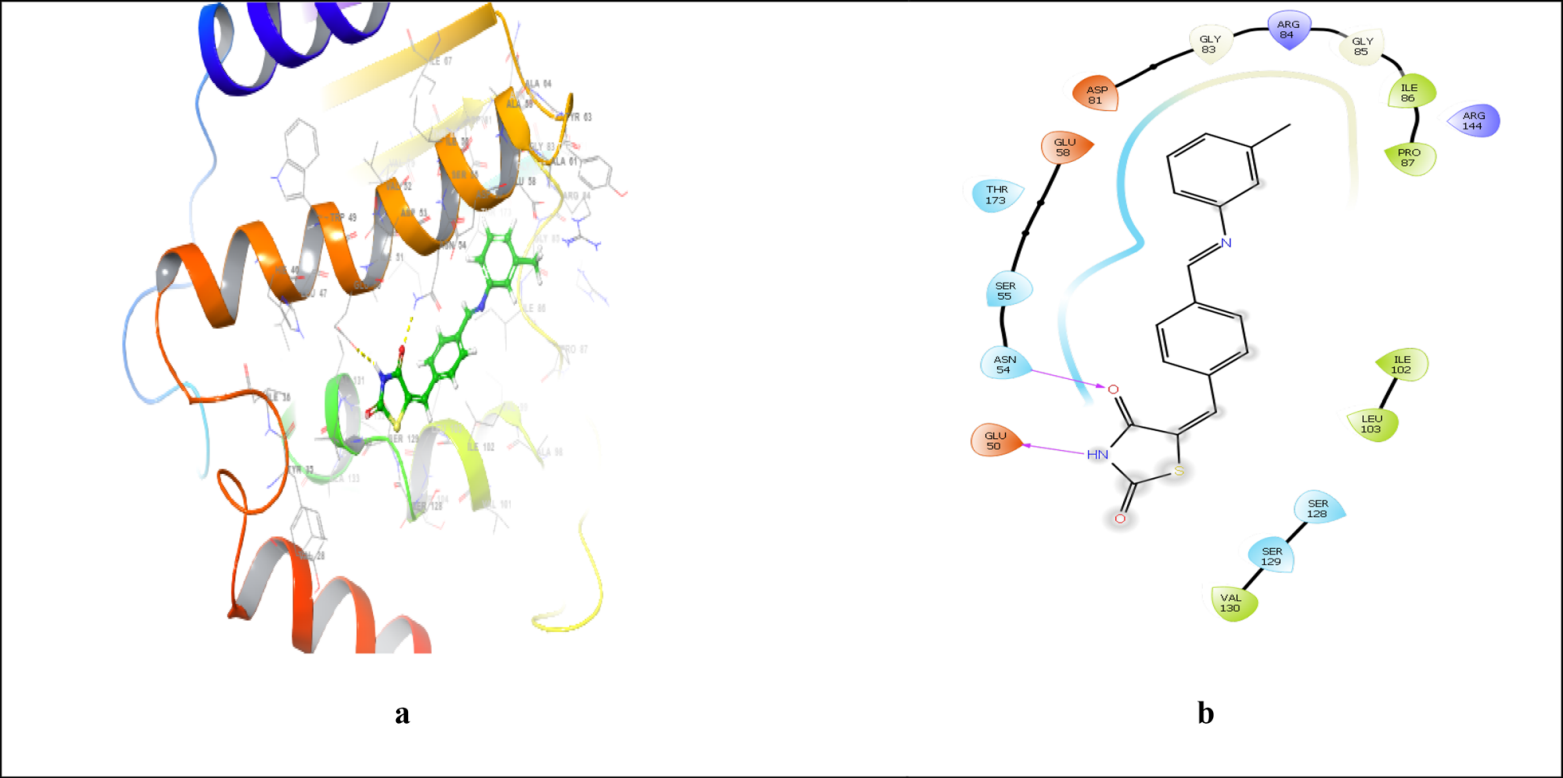
Interaction of compound **7** and 4-bromo-5-methyl-*N*-[1-(3-nitropyridin-2-yl)piperidin-4-yl]-1*H*-pyrrole-2-carboxamide within the active pocket of *S. aureus* GyrB ATPase domain protein and interacting amino acid in 3D (**a**) and 2D (**b**) view

**Fig. 8:**
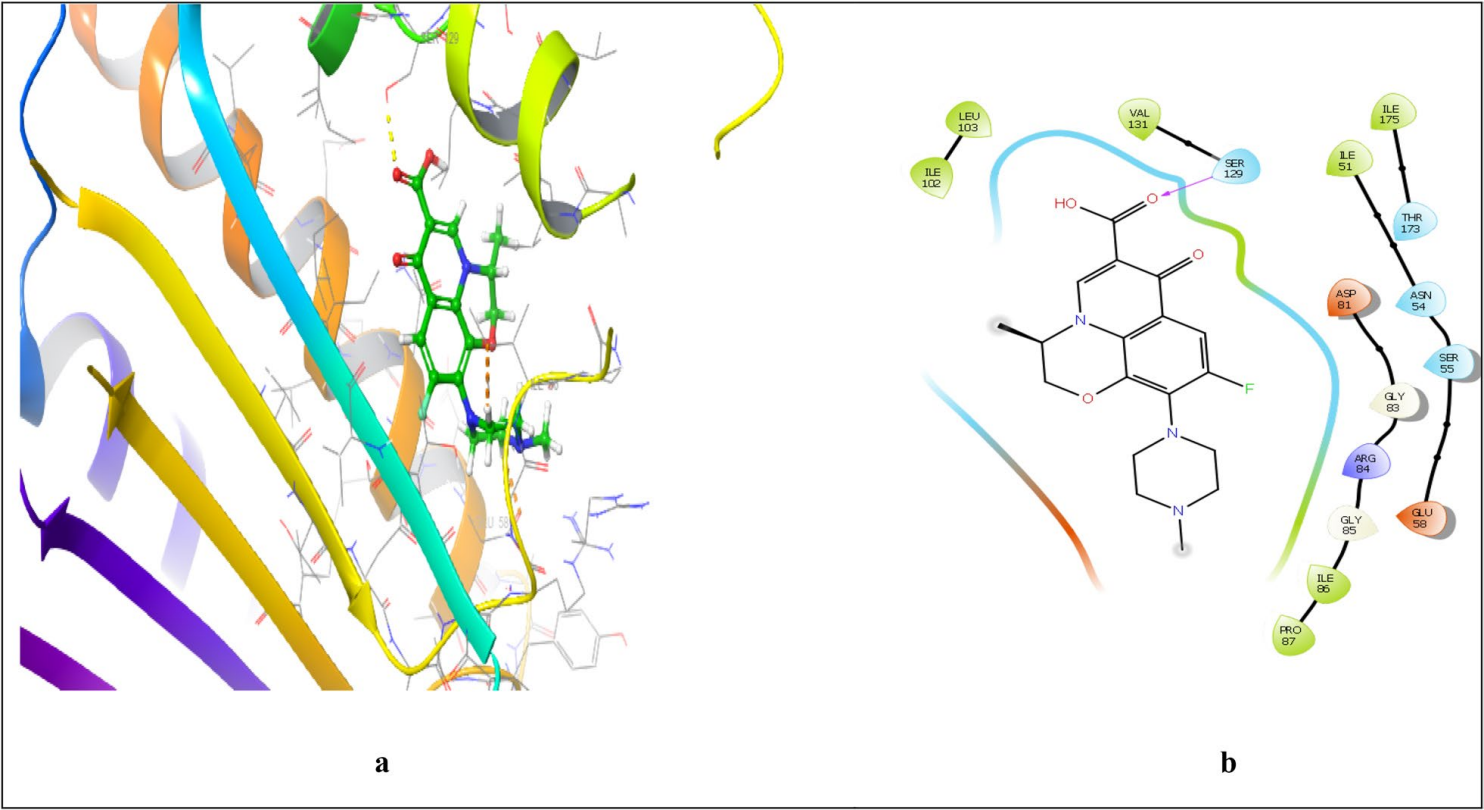
Interaction of standard compound ofloxacin and 4-bromo-5-methyl-*N*-[1-(3-nitropyridin-2-yl)piperidin-4-yl]-1*H*-pyrrole-2-carboxamide within the active pocket of *S. aureus* GyrB ATPase domain protein and interacting amino acid in 3D (**a**) and 2D (**b**) view

Binding affinity of the compounds was demonstrated in terms of binding energy, calculated in term of negative energy. Binding affinity is more when binding energy is less. Docking scores were shown as numerical value of interaction energy which is statistical evaluation function for displaying the results. Different visualization tools were used to visualize the 3D pose of the ligand interaction with receptor [25]. Molecular docking study revealed that the synthesized compounds exhibited good interaction with crucial amino acids of protein. The best-fitted compounds **4** and **7** showed the best docking scores of − 4.73 and − 4.61, respectively in comparison to standard drug ofloxacin (docking score = − 5.107) within the ATP binding pocket (Table 5). Ligand interaction diagram and binding mode of most active compounds **4**, **7** and standard drug ofloxacin in the active site of *S. aureus* GyrB ATPase domain co-crystallized ligand 08B are shown in (Table 5, Figs. 6, 7, 8). Docking studies revealed that antimicrobial compounds having better activity than the standard drug ofloxacin can be obtained by further optimizing the structure of the compounds **4** and **7**.

**Table 5:** Docking score and binding energy of compound **4** and **7** with standard drug ofloxacin

Compound	Docking score	Interacting residues
4	**− **4.73	PRO87, ILE86, GLY85, ARG84, GLY83, ASP81, THR173, GLU 58, SER55, ASN54, ILE51, GLU50, VAL131, VAL130, SER129, SER128, LEU103, ILE102
**7**	**− **4.61	PRO87, ARG144, ILE86, GLY85, ARG84, GLY83, ASP81, THR173, GLU 58, SER55, ASN54, GLU50, VAL130, SER129, SER128, LEU103, ILE102
Ofloxacin	**− **5.107	PRO87, ILE86, GLY85, ARG84, GLY83, ASP81, VAL131, SER129, SER128, LEU103, ILE102, THR173, ILE175, GLU58, SER55, ASN54, ILE51

### ADME results

QikProp module of Schrodinger 2018-1 (Maestro version *11.5*) was used for studying ADME parameters of the synthesized molecules. Around eleven pharmacologically significant and physically relevant parameters of the synthesized compounds (**1–20**) were studied. The ADME results of the synthesized TZD analogues **1–20** exhibited fairly good results within the recommended range of Qikprop module and in good agreement with Lipinski’s rule of five which includes Molecular weight of the molecule (mol. MW = < 500), Predicted water/gas partition coefficient (QPlogKp = − 8.0 to − 1.0), Percent human oral absorption (0 to 100), donor HB (0.0 to − 6.0), (QPlogPw = 4.0 to − 45.0), Predicted octanol/water partition coefficient (QPlogPo/w = − 2.0 to − 6.5), human oral absorption (1, 2 or 3), Predicted brain/blood partition coefficient (QPlogBB = − 3.0 to − 1.2), accept HB (2.0 to − 20.0) and hence found these analogues as suitable drug candidates. The results of ADME studies are expressed in the Table 6.

**Table 6:** ADME parameters of synthesized compounds

Comp.	ADME parameters										
	Mol MW	Rule of Five	QPlogPo/w	Human Oral Absorption	Volume	% Human Oral Absorption	QPlogP_w_	QPlogK_p_	QPlogBB	Donor HB	Accept HB
1	308.354	0	3.38	3	957.885	100.0	8.709	**− **1.987	**− **0.778	1.0	4.0
**2**	247.271	0	0.656	3	743.851	67.916	11.506	**− **4.204	**− **1.339	3.0	5.0
**3**	323.369	0	2.789	3	999.18	91.672	11.575	**− **2.168	**− **1.011	2.0	5.5
**4**	342.799	0	3.817	3	995.909	100.0	8.468	**− **2.092	**− **.0.613	1.0	4.0
**5**	342.799	0	3.871	3	1001.91	100.0	8.469	**− **2.155	**− **0.631	1.0	4.0
**6**	312.342	0	2.331	3	947.57	91.759	9.026	**− **2.078	**− **0.808	1.0	5.0
**7**	322.381	0	3.687	3	1017.578	100.0	8.404	**− **2.183	**− **0.813	1.0	4.0
**8**	322.381	0	3.688	3	1017.852	100.0	8.405	**− **2.184	**− **0.814	1.0	4.0
**9**	336.408	0	3.986	3	1072.652	100.0	8.091	**− **2.333	**− **0.807	1.0	4.0
**10**	336.408	0	3.958	3	1063.481	100.0	8.109	**− **2.259	**− **0.758	1.0	4.0
**11**	326.344	0	3.583	3	971.486	100.0	8.509	**− **2.081	**− **0.674	1.0	4.0
**12**	387.25	0	3.949	3	1010.987	100.0	8.477	**− **2.157	**− **0.622	1.0	4.0
**13**	353.352	0	2.681	3	1031.006	77.031	9.831	**− **3.889	**− **1.84	1.0	5.0
**14**	387.797	0	3.239	3	1073.076	82.122	9.524	**− **3.85	**− **1.579	1.0	5.0
**15**	338.38	0	3.509	3	1036.846	100.0	8.946	**− **2.059	**− **0.866	1.0	4.75
**16**	338.38	0	3.475	3	1032.883	100.0	8.937	**− **2.085	**− **0.869	1.0	4.75
**17**	338.38	0	3.477	3	1033.285	100.0	8.943	**− **2.086	**− **0.872	1.0	4.75
**18**	326.344	0	3.615	3	974.01	100.0	8.488	**− **2.121	**− **0.674	1.0	4.0
**19**	353.352	0	2.677	3	1030.425	77.006	9.824	**− **3.892	**− **1.835	1.0	5.0
**20**	400.578	0	5.921	1	1434.834	100.0	6.164	**− **1.791	**− **1.671	1.0	4.5

#### Structure activity relationship

**Fig. 9:**
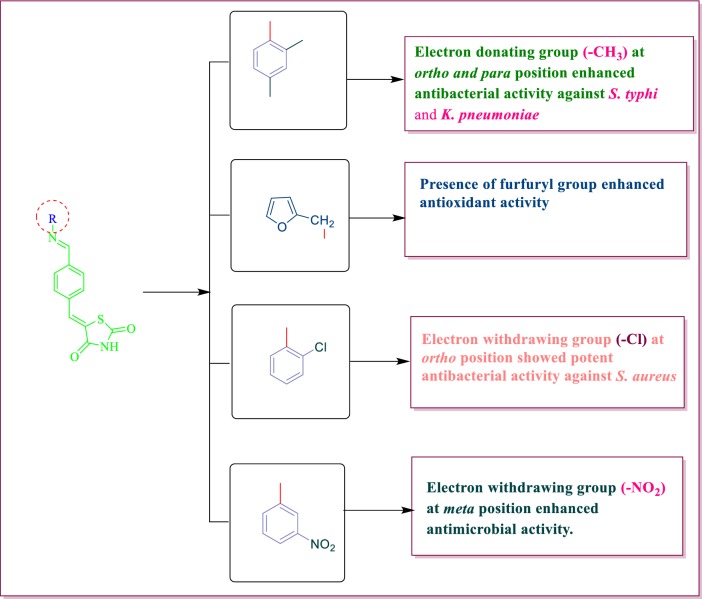
Structure activity relationship of synthesized compounds

From the antimicrobial and antioxidant evaluation studies following structure activity relationship can be drawn (Fig. 9):

- The different substitution of amines/anilines used to synthesize the final derivatives of 5-((*E*)-4-((*E*)-(substitutedaryl/alkyl)methyl)benzylidene)thiazolidine-2,4-dione played an important role in improving the antimicrobial and antioxidant activities. Substitution of electron releasing group methyl (–CH_3_) at *ortho* and *para* position in the synthesized compound **9**, increased the antibacterial potential against *S. typhi and K. pneumoniae.*
- Presence of Electron withdrawing group nitro (–NO_2_) at *meta* position enhanced antibacterial potential against *K. pneumoniae* and *S. aureus* as well antifungal activity against *C. albicans* and *A. niger* (Compound **4**).
- Presence of electron withdrawing group fluoro (F) at *ortho* position of the synthesized compound **11,** enhanced the antibacterial potential against *E. coli* whereas substitution of heterocyclic group furfuryl in the derived compound **6,** improved the antioxidant potential. These molecules may further be used as lead compounds to derive more potent and less toxic novel antioxidant and antimicrobial agents.

## Conclusion

A series of thiazolidine-2,4-dione derivatives was designed, derived and then evaluated for its antioxidant and antimicrobial evaluations. The biological screening outcomes indicated that the molecules **4**, **9**, **11**, **12, 13**, **15** and **16** showed promising activity against the selected strains of microbial species. Antioxidant evaluation found compound **6** having IC_50_ = 9.18 μg/mL to be the most potent compound. Further, the molecular docking evaluation was carried out to find out the interaction between synthesized thiazolidine-2,4-dione compounds with DNA gyrase protein indicated that compound **4** (Docking score = − 4.73) and **7** (Docking score = − 4.61) showed better potency within the ATP binding pocket by showing good dock score and hence can further be used as lead structures for rationally designing the antimicrobial molecule.

### Experimental

The chemicals of analytical grade were procured from commercial sources and were used for the synthesis without any purification. Open glass capillaries on a Stuart scientific SMP3 apparatus were used for determining melting point (m.p.) and reported uncorrected. Reaction progress was monitored by TLC (Thin layer chromatography) glass plates of silica gel G for every synthetic step. KBr pellets were used for recording infrared (IR, KBr, cm^−1^) on Bruker 12060280 (Software: OPUS 7.2.139.1294) spectrophotometer. Bruker Avance III 400 NMR spectrometer was used to determine ^1^H spectra in appropriate deuterated solvents and using tetramethylsilane as internal standard and are expressed in parts per million (*δ*, ppm) downfield from internal standard. Mass spectra was obtained using Waters Micromass Q-ToF Micro instrument. CHN analyzer was used to perform elemental analysis.

### Synthetic steps of Scheme 1

#### Step 1: Synthesis of thiazolidin-2,4-dione TZD (**I**)

To a solution of chloroacetic acid (0.06 mol) in water (15 mL), thiourea (0.06 mol) in water (15 mL), acid was added and stirred till the occurrence of white precipitate. To the contents of flask, 6 mL of conc. HCl was added dropwise followed by refluxing for 10 h. On cooling, needle shaped crystals of TZD (**I**) were obtained which were filtered, dried and recrystallized using methanol as solvent [6].

#### Step 2: Synthesis of 4-((2,4-dioxo-1,3-thiazolidin-5-ylidene)methyl)benzaldehyde (**II**)

To a solution of (**I**) (0.03 mol) and terephthalaldehyde (0.03 mol) in ethanol (45 mL), 3 mL of piperidine (0.0188 mol) was added, stirred and refluxed for next 12 h. Contents of flask were then poured on ice followed by acidification with acetic acid (glacial). Yellow coloured product of 4-((2,4-dioxo-1,3-thiazolidin-5-ylidene)methyl)benzaldehyde (**II**) was obtained which was filtered, dried and further recrystallized using ethanol as solvent [26].

#### Step 3: Synthesis of various title compounds (**1–20**)

To the solution of compound **II** (0.01 mol) in methanol (50 mL), different substituted amines (0.01 mol) were added using catalytic amount of acetic acid (glacial) and refluxed for 4–18 h. The reaction mixture was then allowed to cool and finally recrystallized from methanol to give final compounds (**1–20**).

### In vitro antimicrobial evaluation

The antimicrobial potential of the synthesized compounds was evaluated by serial tube dilution method [27] using fluconazole (antifungal) and cefadroxil (antibacterial) as standard drugs. Both Gram +ve {MTCC-3160 (*S. aureus*), MTCC-441 (*B. subtilis*)} and Gram −ve {MTCC-3231 (*S. typhi*), MTCC-9024, (*K. pneumoniae*) and MTCC-443 (*E. coli*)} bacterial species were used in the study. The antifungal potential was evaluated against MTCC-281 (*A. niger*) and MTCC-227 (*C. albicans*) strains. Nutrient broth double strength I.P. (for bacteria) or sabouraud dextrose broth I.P. (for fungi) [28] nutrient media were used for antimicrobial potential. Stock solutions of the test and reference compounds were prepared in dimethyl sulfoxide. A control set was also used at the same dilutions with the test medium supplemented with dimethyl sulfoxide. Results were recorded in MIC after incubating the samples at 25 ± 1 °C (7 days) for *A. niger*, at 37 ± 1 °C (24 h) for bacteria and at 37 ± 1 °C (48 h) for *C. albicans*, respectively. MIC was recorded for the tested compound as lowest concentration that showed no observable growth of microorganisms in the test tube.

### In vitro antioxidant assay

The antioxidant evaluation of synthesized thiazolidine-2,4-dione derivatives was determined using stable 2, 2-diphenyl-1-picrylhydrazyl (DPPH) free radical scavenging model [29]. The diluted solution of synthesized compounds in methanol of 25 μg/mL, 50 μg/mL, 75 μg/mL and 100 μg/mL were prepared and equal amount of methanolic solution of DPPH (0.0039%) was added followed by vigorous shaking. The above solution was then kept in dark for 30 min and absorbance of the solution was measured spectrophotometrically at 517 nm using UV–visible double beam spectrophotometer. The mean of at least three observations was taken as mean IC_50_ value in the data presented.

### Molecular docking study

The target protein for thiazolidine-2,4-dione derivatives was identified through the literature. *S. aureus* GyrB ATPase (PDB Id: 3U2D) co-crystallized with 08B ligand, an excellent target for docking against *S. aureus* strain [30] was retrieved from Protein Data Bank (http://www.rcsb.org/pdb/home/home.do) to dock synthesized thiazolidine-2,4-dione compounds. Docking score was obtained from GLIDE software through targeted the ATP binding site by creating active site grid. The active site grid possessed the important amino acids which interact with ATP [31].

## Data Availability

We have presented all our main data in the form of tables and figures.

## Abbreviations

ADME: Absorption, distribution, metabolism and excretion
μM: Micro mole
PDB: Protein data bank
TZD: Thiazolidin-2,4-dione
H-NMR: Proton nuclear magnetic resonance
FT-IR: Fourier transform infrared
MS: Mass spectrometry
DPPH: 2,2-Diphenyl-1-picrylhydrazyl
DNA: Deoxyribonucleic acid
ATP: Adenosine triphosphate
MRSA: Multidrug resistant *Staphylococcus aureus*
VRE: Vancomycin-resistant *enterococci*
MIC: Minimum inhibitory concentration
GyrA: DNA gyrase subunit A
GyrB: DNA gyrase subunit B
TLC: Thin layer chromatography
IC_50_: Half maximal inhibitory concentration
